# Supplementary figures and images for: Tetraspanin Cd9b and Cxcl12a/Cxcr4b have a synergistic effect on the control of collective cell migration
Source: PLoS One. 2021 Nov 30;16(11):e0260372. doi: 10.1371/journal.pone.0260372 (PMC8631670; doi:10.1371/journal.pone.0260372)

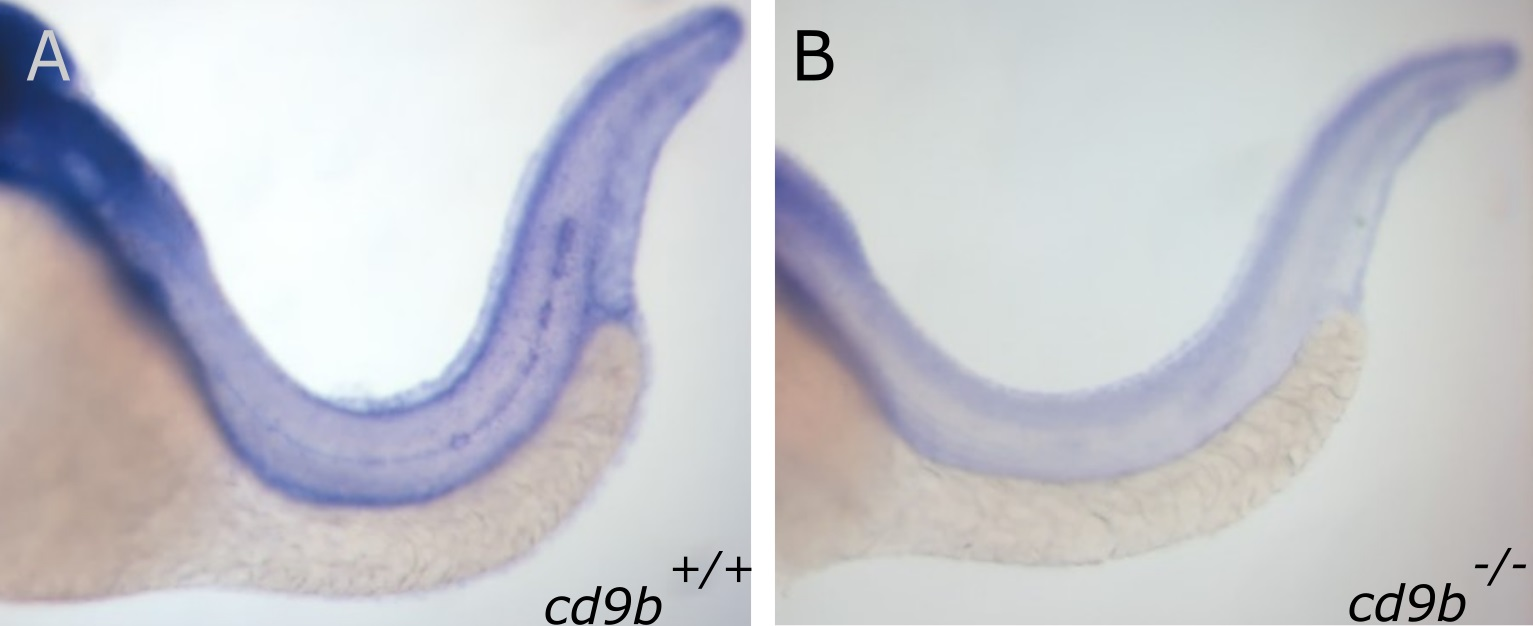

Supplement: S1 Fig — A-B: Representative images of cd9b WISH at 36 hpf in (a) WT and (b) cd9b homozygous embryos. (TIF) [file pone.0260372.s001.tif]

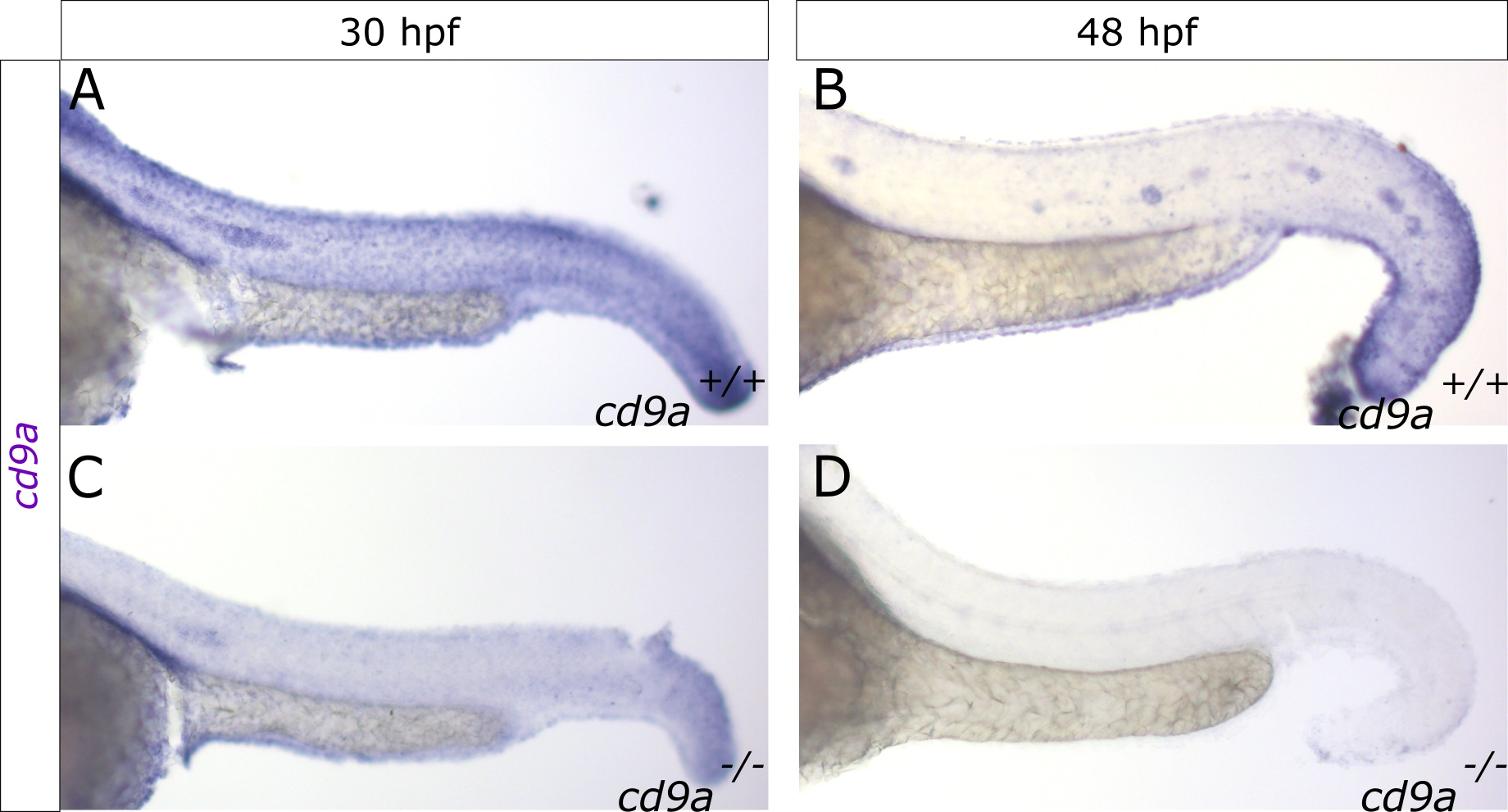

Supplement: S2 Fig — A-D: Representative images of cd9a WISH on (a-b) WT and (c-d) cd9a homozygous embryos at time shown. (TIF) [file pone.0260372.s002.tif]

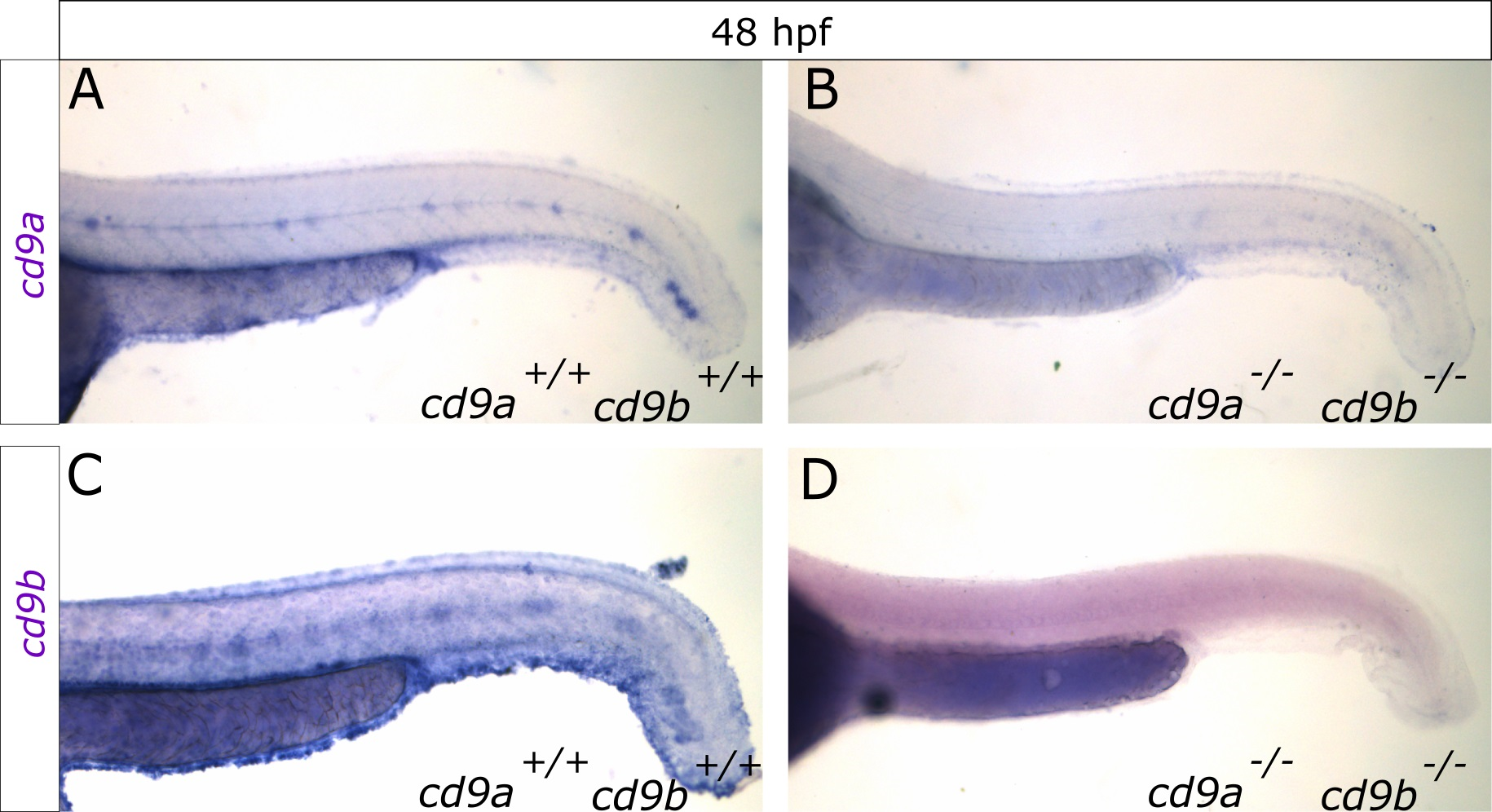

Supplement: S3 Fig — A-B: Representative images of cd9a ISH on (a) WT and (b) cd9 dKO mutants at time shown. C-D: Representative images of cd9b ISH on (c) WT and (d) cd9 dKO mutants at time shown. (TIF) [file pone.0260372.s003.tif]

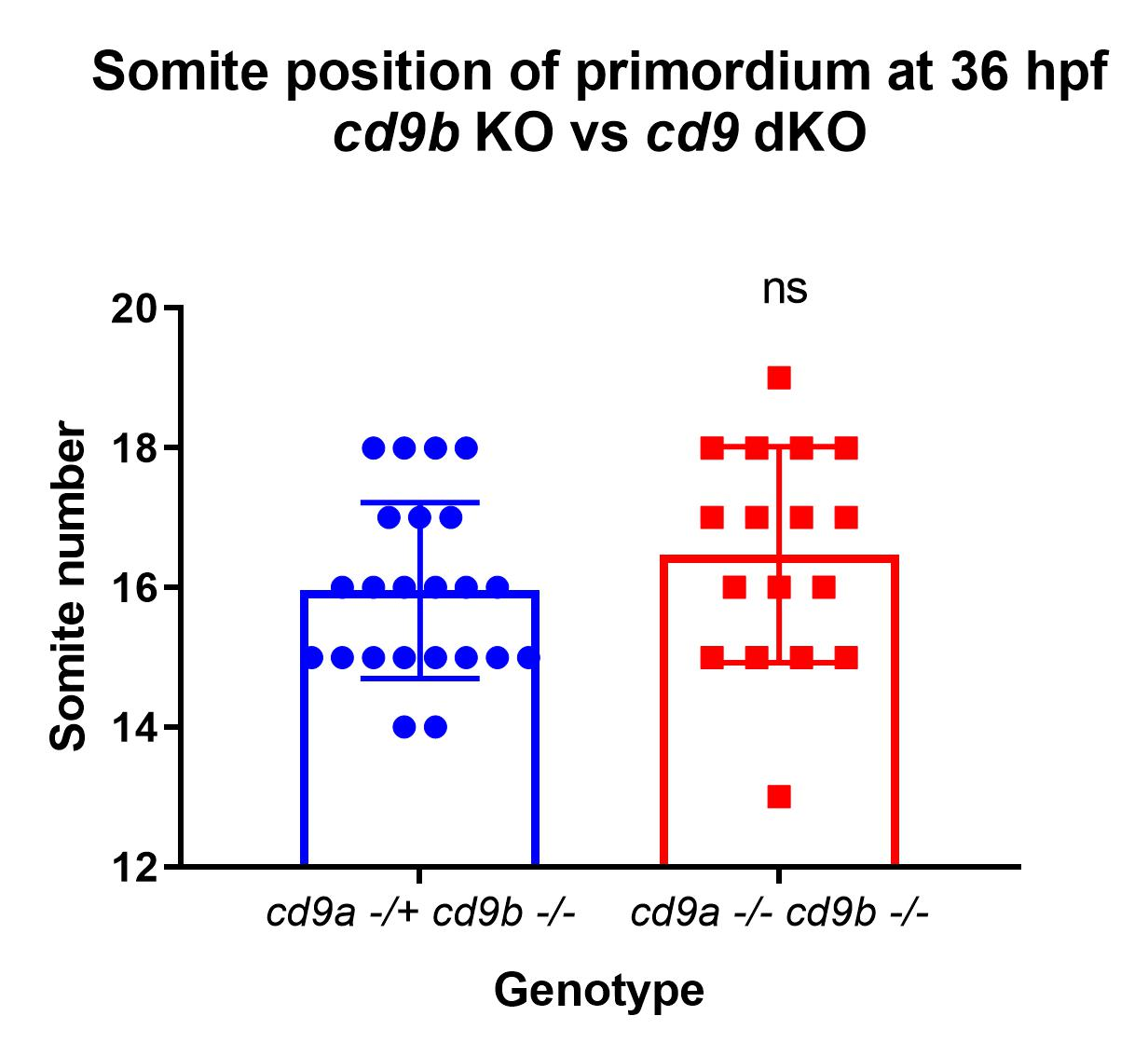

Supplement: S4 Fig — Graph showing the distance migrated by the primordium at 36 hpf is the same in cd9b KO and cd9 dKO embryos. Significance was assessed using an unpaired t test, N = minimum 17. Bars show mean +/- SD. (TIF) [file pone.0260372.s004.tif]

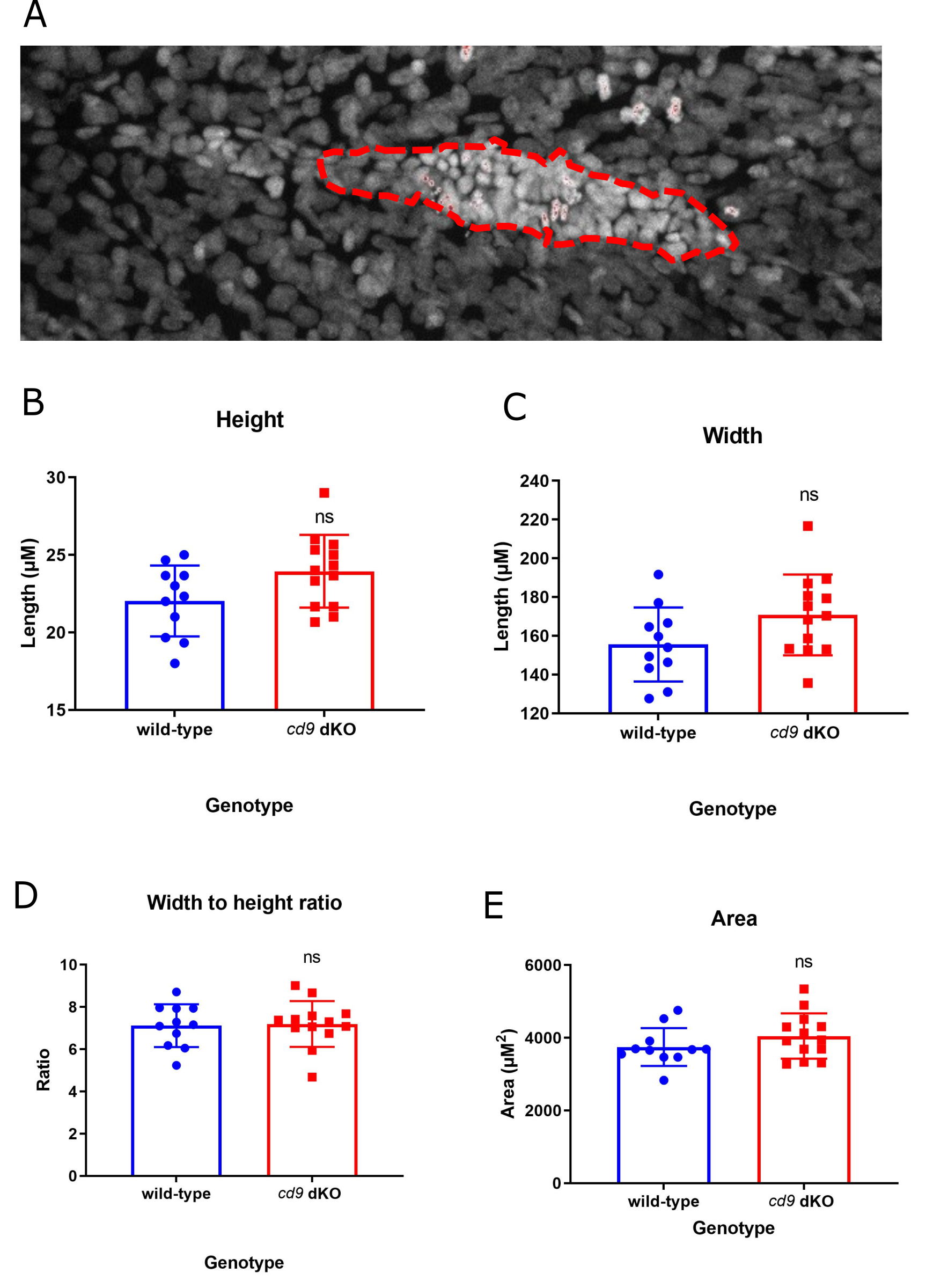

Supplement: S5 Fig — A: Representative image of a DAPI-stained primordium from a WT zebrafish at 36 hpf with red outline to show measured area. B-E: Graphs showing measurements of the primordium (b) height, (c) width, (d) width to height ratio, and (e) area in WT and cd9 KO embryos. The height was measured three times at equal points along the primordium and then averaged. The width of primordium was measured from the two furthest points along the middle of the primordium. Width was then divided by height to generate a ratio. The area was circled using the freehand selection and measured. Cells were counted using the multipoint tool on Image J software. Significance was assessed using an unpaired t test, p = <0.05, N = minimum 11. Bars show mean +/- SD. (TIF) [file pone.0260372.s005.tif]

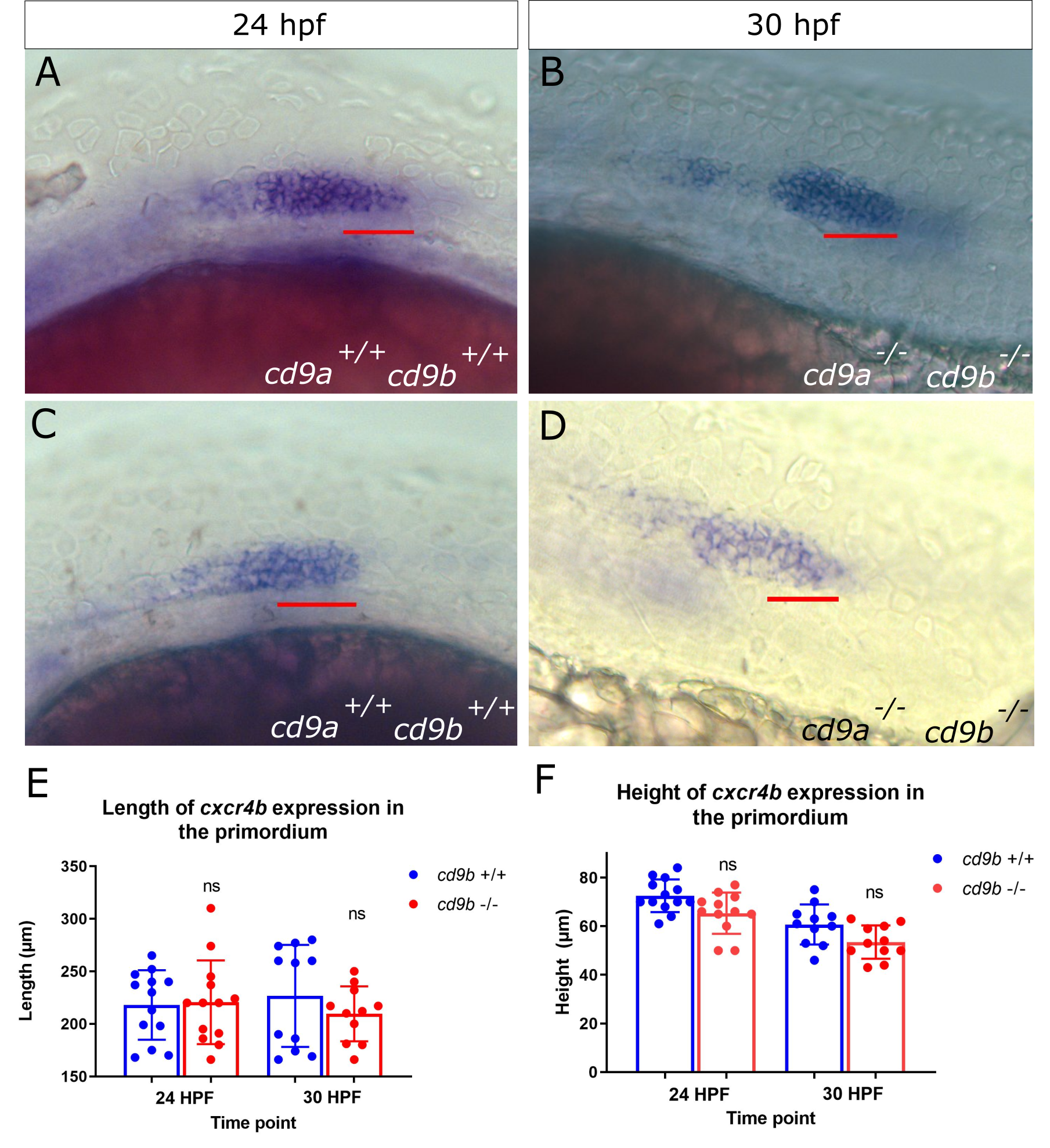

Supplement: S6 Fig — A-D: Representative images of cxcr4b ISH in the primordium of (a,c) WT and (b,d) cd9 dKO mutants at time shown. Scale bar: 50 μm. E-F: Graphs showing measurements of (e) length and (f) height of cxcr4b expression in the primordium of WT and cd9 KO embryos at indicated stages. Length measurements were taken along the middle of the embryo between the two furthest points of expression within the primordium. Height measurements were taken between the two highest points of expression within the primordium. Significance was assessed using an unpaired T test. N = minimum 13 for 24 hpf and 11 for 30 hpf. Bars show mean +/- SD. (TIF) [file pone.0260372.s006.tif]

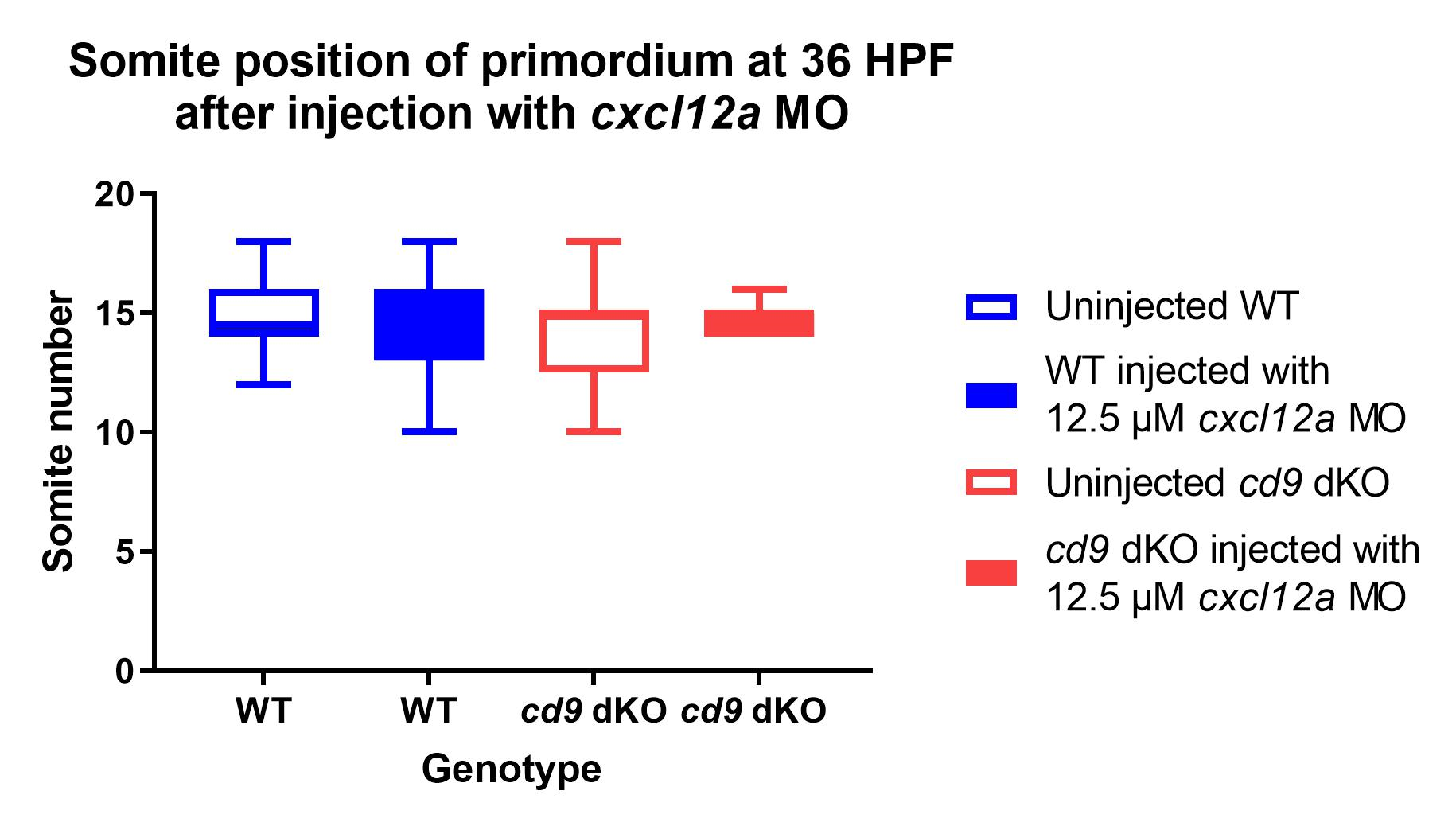

Supplement: S7 Fig — Distance migrated by the primordium (labelled by claudin b ISH) was recorded for injection of 12.5 μM cxcl12a MO. Significance was assessed using one-way ANOVA. N = minimum 13. Box extends from the 25th to 75th percentile and whiskers from 10th to 90th. (TIF) [file pone.0260372.s007.tif]
