# Supplementary material for: Tetraspanin Cd9b and Cxcl12a/Cxcr4b have a synergistic effect on the control of collective cell migration
Source: PLoS One. 2021 Nov 30;16(11):e0260372. doi: 10.1371/journal.pone.0260372 (PMC8631670; doi:10.1371/journal.pone.0260372)
Supplement: Attachment — Submitted filename: PlosOne Review_Phan Quang Tien.docx [file pone.0260372.s010.docx]

In this manuscript, the authors describe the involvement of *cd9a* and *cd9b* in the collective cell migration of posterior lateral line primordium and the neuromast formation in zebrafish. Also, authors show that disruption of Cxcl12a/Cxcr4b signaling could further delay the primordium migration in *cd9a* and *cd9b* double mutant. Overall, this work continues the work of Gallardo et al., 2010 and provides more function of both *cd9a and cd9b* in the primordium migration and neuromast formation.

However, the manuscript contains plenty of errors and most of the figures do not show all images/panels described in the text. At this stage, the manuscript is not competent for publication and should be prepared again with more attention.

Here are some examples of errors and mistakes that impede the review process:

**Major errors:**

Line 159-161 and Figure 1: mislabeling cd9a (DEF), cd9b (ABC)

Line 173 – 175 and Figure 2: Only Figure 2A-B-C-D are shown and they are not in line with the description in the text.

Figure 3A: cd9a is mislabelled at the top left panel of the intron-exon graphic.

Figure 4 and Figure 5 contain only images/panels A-B-C-D-E-F-G; panels I-J are missing while being described in the text.

**Some examples of many minor errors:**

Throughout the manuscript, the use of upper and lower case letters to label protein symbols for fish and for human should be corrected according to standard gene/protein nomenclature. For example, lines 22, 28, etc., only the first letter of protein symbols should be in upper-case for fish.

Line 23: add a fullstop after “for this process”

Line 34: reference format

Line 74: reference format

Line 154: “MUSCLE” should be spelled out

Figure 1: Image in A needs a different scale bar as it appears that the image in D is of different magnification than image in A.

Figure 1: One image of the sense probes of cd9a and cd9b should be added.

Figure 1 B and E: The embryo images in B and E appear younger than 48 hpf. It would be more demonstrative if authors can show alternative images for this time point. It’s known that pLLP migration is complete by 48 hpf with the terminal cluster of NM deposited at the tail end. This is not clearly observed in embryo in B. Although the image in A shows the labelling of primordium structure, a colocalisation of cd9 in situ with a known marker of neuromast (such as *ath1 or cldnb)* would strongly support the author’s statement that cd9 genes are expressed in deposited neuromasts.
