# Supplementary material for: Tetraspanin Cd9b and Cxcl12a/Cxcr4b have a synergistic effect on the control of collective cell migration
Source: PLoS One. 2021 Nov 30;16(11):e0260372. doi: 10.1371/journal.pone.0260372 (PMC8631670; doi:10.1371/journal.pone.0260372)
Supplement: Attachment — Submitted filename: Response to Reviewers v1.docx [file pone.0260372.s011.docx]

Christoph Winkler, Dr.

Academic Editor

PLOS ONE

Manuscript no. PONE-D-21-17584

Manuscript title: Augmentation of chemokine signalling by CD9 tetraspanins facilitates collective cell migration

Dear Dr Winkler,

We were glad to learn that the reviewers found our work interesting and that, pending revisions, the manuscript is acceptable for publication in PLOS ONE. We modified our manuscript according to the comments of the reviewers as detailed in the point-by-point response below. The modified manuscript has highlighted modified text in red. We thank the reviewers for their constructive criticisms and hope that you will find the manuscript suitable for publication in PLOS ONE.

Sincerely,

Dr. Katherine Marsay

**Detailed response to comments:**

We would like to thank the reviewers for their constructive comments that helped us to improve the manuscript. As detailed below we modified the manuscript according to these comments.

Journal Requirements

**Comment**: 1. Please ensure that your manuscript meets PLOS ONE's style requirements.

**Answer**: High attention was made to ensure all PLOS ONE style requirements were met including: correct formatting in corresponding author and joint author information, correct acknowledgements, correct reference format.

**Comment:** 2. PLOS requires an ORCID iD for the corresponding author in Editorial Manager on papers submitted after December 6th, 2016.

**Answer**: HR has validated his ORCID iD.

**Comment:** 3. We note that you have included the phrase “data not shown” in your manuscript. Unfortunately, this does not meet our data sharing requirements.

**Answer:** Previous results referred to as “data not shown” have been added to Supplementary figures S5 and S6.

Reviewer #1:

**Comment:** The manuscript contains plenty of errors and most of the figures do not show all images/panels described in the text.

**Answer:** All figures were evaluated in detail and corrected to match figure legends. Gene and protein labels were corrected.

**Comment**: Throughout the manuscript, the use of upper and lower case letters to label protein symbols for fish and for human should be corrected according to standard gene/protein nomenclature.

**Answer**: All fish and human gene and protein labels have been corrected according to standard gene/protein nomenclature.

**Comment**: reference format e.g. line 34

**Answer**: All references were checked and reformatted correctly.

**Comment**: There were a few comments regarding figure 1 so I will address them together:

Image in A needs a different scale bar as it appears that the image in D is of different magnification than image in A. One image of the sense probes of cd9a and cd9b should be added. The embryo images in B and E appear younger than 48 hpf. It would be more demonstrative if authors can show alternative images for this time point. Although the image in A shows the labelling of primordium structure, a colocalization of cd9 in situ with a known marker of neuromast (such as ath1 or cldnb) would strongly support the author’s statement that cd9 genes are expressed in deposited neuromasts.

**Answer**: Figure 1 was redrawn to best illustrate *cd9a* and *cd9b* expression in the lateral line. For 36 hpf and 48 hpf a low and high magnification image is included so the expression can be seen in the posterior lateral line as a whole, as well as in detail in the primordium or neuromasts. The images of the neuromasts at high magnification show more clearly how *cd9* is specifically expressed in the cell membranes of most cells in a deposited neuromast and supports the statement that both *cd9* paralogues are expressed in deposited neuromasts. Scale bars are consistently 50 µM throughout the figure. As requested, one image of the sense probes of *cd9a* and *cd9b* were added.

Reviewer #2:

**Comment**: While strictly speaking it may be correct to say the function is less “robust”, the analysis is minimal and it is remains unclear what the role of cd9 in chemokine might be or whether the exaggeration of deficits seen specifically in the double mutants necessarily result from a role of cd9 in determining efficacy of chemokine signaling.

**Answer**: Our morpholino results show that a lack of Cd9 exacerbates *cxcr4b* knockdown and primordium delay. The reviewer is correct that we cannot assign a specific role of Cd9 to Cxcr4b in this pathway. We changed some wording throughout our manuscript to support our results while keeping the potential mechanisms of Cd9 broad. e.g.

New title: Augmentation of chemokine signalling by Cd9 tetraspanins facilitates collective cell migration.

Abstract: Together these results provide evidence that Cd9 modulates collective cell migration of the pLLP during zebrafish development, potentially through promoting Cxcxr4b signalling.

**Comment**: The deficits seen in cd9 double mutants are not typical of those seen when chemokine signaling alone is compromised as it is not even clear whether primordium migration is slower or simply delayed in its initiation. A deficit in chemokine signaling alone might be expected to slow migration and reduce spacing between deposited neuromasts, however, this is not what is seen in the mutants.

**Answer**: We did not wish to make the claim that Cd9 is acting solely on Cxcr4 signalling and apologize if this was not made clear. We have now been more explicit about this in the discussion and explained that we chose to investigate Cxcr4b/Cxcl12 signaling as CD9 has been previously reported to interact and regulate with this signaling pathway in the migration of mammalian cells.

**Comment**: Furthermore, as collective migration of the primordium is not just dependent on chemokine signaling but also, at a minimum, on Fgf signaling, it is not clear if the observed deficits in migration seen specifically in cd9 mutants might also be seen when Fgf signaling dependent migration is compromised. The authors should test if compromised migration, observed in cd9 double mutants in the background of compromised cxcr4b/cxcl12a function, is also seen when migration is slowed with subcritical doses of an Fgf signaling inhibitor. Are synergistic effects on migration only seen following manipulation of chemokine dependent migration and not following subcritical interference with Fgf-dependent migration?

**Answer**: The reviewer is correct that we failed to discuss other potential mechanisms of Cd9 inhibition that could result in a delayed migration. We expanded the discussion to review other signalling mechanisms that could be relevant to Cd9 such as the regulation of Wnt signalling pathways. Cd9 has been previously reported to downregulate this pathway in cell culture. Unfortunately, I am no longer in a position to perform experiments on the mutant fish but the reviewer provoked an interesting discussion for future investigation.

**Comment:** The literature suggests quite a broad range of potential roles for Cd9 and it would be premature to conclude that changes following loss of cd9b function demonstrated in this study are necessarily related to a role for CD9 in facilitating chemokine signaling.

**Answer:** The reviewer is correct that we failed to discuss other mechanisms aside from chemokine signalling that could be associated with tetraspanins such as Cd9. We expanded the discussion to review other tetraspanin related functions that could be affecting cell migration in the developing primordium such as Cadherin-dependent intercellular adhesion.
