# Supplementary material for: Tetraspanin Cd9b and Cxcl12a/Cxcr4b have a synergistic effect on the control of collective cell migration
Source: PLoS One. 2021 Nov 30;16(11):e0260372. doi: 10.1371/journal.pone.0260372 (PMC8631670; doi:10.1371/journal.pone.0260372)
Supplement: Attachment — Submitted filename: Response to Reviewers v1 05112021.docx [file pone.0260372.s012.docx]

Christoph Winkler, Dr.

Academic Editor

PLOS ONE

Manuscript no. PONE-D-21-17584

Manuscript title: Cd9b tetraspanin and Cxcl12a/Cxcr4b have a synergistic effect on the control of collective cell migration

Dear Dr Winkler,

We appreciate that you saw the merit in our research and we were happy to incorporate the minor revisions according to the comments of the reviewers. They are detailed in the point-by-point response below and the modified manuscript has highlighted modified text in red. Previous revisions required additional experiments that were performed by Harsha Mahabaleshwar and Charmaine Ho Min. The main authors who performed the majority of the research are now in positions where they are unable to access the facilities associated with this research. Harsha and Charmaine aided the communication and organisation of the revisions between the authors despite us all being in different continents. They were vital for the latest version of the manuscript and therefore, they were added as additional authors to this manuscript.

We thank you and the reviewers for your patience and for giving us another chance to meet the publication criteria of PLOS ONE. We hope that you will find the revised manuscript suitable for publication in PLOS ONE.

Sincerely,

Dr. Katherine Marsay

**Detailed response to comments:**

We would like to thank the reviewers for their constructive comments that helped us to improve the manuscript. As detailed below we modified the manuscript according to these comments.

Journal Requirements

**Comment**: Please review your reference list to ensure that it is complete and correct.

**Answer**: All references were reviewed thoroughly to ensure they were complete, correct and that none had been retracted since the first draft of this manuscript. Two references were added in response to a comment by reviewer 2 as detailed later. Reference 15 was noticed as an incorrect reference and replaced from Charrin et al., 2003 to Ovalle et al., 2007. Reference 42 was noticed to be a duplication of reference 16 and deleted. No other changes to the reference list were required.

Reviewer #1:

**Comment:** The title “Augmentation of chemokine signalling by Cd9 tetraspanins facilitates collective cell migration” is missleading as there is no direct data showing that Cd9 enhances Cxcl12/Cxcr4 expresion or activity which in turn facilitates collective cell migration. The authors may consider a more direct title, for instance “Cd9b tetraspanin and Cxcl12a/Cxcr4b convey synergistic effect on the control of collective cell migration”.

**Answer:** I agree that although our data may provide clues for future research, our current observations on their own are not adequate to suggest a conclusive interaction between Cd9b and the Cxcl12/Cxcr4 pathway in the control of primordium migration. I appreciate the suggested alternative title and would be pleased to use a slightly modified version of this as the new title for our manuscript.

**Comment:** Line 311: typo “Cxcrb” change to “Cxcr4b”

**Answer:** Changed.

Reviewer #2:

**Comment:** The title should be changed. A subtly different but perhaps less misleading title could be: “Loss of Cd9 exaggerates deficits in collective migration caused by interference with chemokine signaling in the posterior Lateral Line primordium”.

**Answer:** I agree that although our current hypothesis is that Cd9 modulates collective cell migration of the pLLP through promoting Cxcr4b signaling, we cannot conclusively prove this from our current results. I appreciate the suggested alternative title but have decided to use the following as the new title for our manuscript: “Cd9b tetraspanin and Cxcl12a/Cxcr4b have a synergistic effect on the control of collective cell migration”.

The loss of Cd9b causes a significant delay in collective migration of the posterior lateral line, **and also** exaggerates deficits in collective migration caused by interference with chemokine signaling. I believe the chosen title describes the phenotype of *cd9b* KO as being similar to that of loss of Cxcr4b and also highlights the interaction I hypothesized from our preliminary chemokine interactions. I hope you agree but I am happy to change the title again if it is still not clear.

**Comment**: Similarly, the abstract should be edited to simply remove or edit the last line: “Loss of both Cd9a and Cd9b sensitized embryos to reduced Cxcr4b and Cxcl12a levels. Together these results provide evidence that Cd9 modulates collective cell migration of the pLLP during zebrafish development.”
OR
“Loss of both Cd9a and Cd9b sensitized embryos to reduced Cxcr4b and Cxcl12a levels. Together these results provide evidence that Cd9 modulates collective cell migration of the pLLP during zebrafish development. One interpretation of these observations is that Cd9 contributes to more effective chemokine signaling.”

**Answer:** I appreciate the suggestions and agree that the last line of the abstract should be edited to make the conclusions clearer and prevent perpetuating overconfident interpretations of results. The abstract was edited in accordance to the latter suggestion as so: “Together these results provide evidence that Cd9 modulates collective cell migration of the pLLP during zebrafish development. One interpretation of these observations is that Cd9 contributes to more effective chemokine signaling.”

**Comment:** One additional issue that requires comment. The video of pLLP migration does not show any obvious deficits in migration and migration speed was never assessed or reported. Is it possible that the difference in position of primordium observed by the authors results from a delay in the start of migration. Do the authors have evidence that argues against this? If not, this possibility should also be raised in the discussion.

**Answer:** The reviewer raises an important point. The delay in primordium migration could either be due to a change in the onset of migration, a change in the speed of migration or a combination of the two. The idea that differences in the observed position of the primordium could be due to a delayed start in migration was added to the discussion. Further experiments that could elaborate on this were also added and involved the addition of two references (44 & 45 - Matsuda et al. 2013, Valdivia et al. 2011). Their publications provide methods that could be used to calculate the speed of primordium migration.

Inserted/edit text is shown below:

Line 387-389: Therefore, the *cd9* dKO phenotype appeared to be a migratory defect that could either be due to a change in the onset of migration, a change in the speed of migration or a combination of the two.

Line 415: We mentioned that the migratory defect could be due to a change in the onset of migration. This could be further investigated by performing *claudin b* ISHs at the onset and earliest stages of development of the posterior lateral line. On the other hand, to try and determine if Cd9b has a role in the speed of migration, ISHs could also be performed at various time points throughout primordium migration and speed could be calculated in a similar method to previous studies [44,45].
